# Comparison of the Performance of Basalt Fiber-Reinforced Composites Incorporating a Recyclable and a Conventional Epoxy Resin

**DOI:** 10.3390/polym17101348

**Published:** 2025-05-15

**Authors:** Farid Taheri, Shahriar Ahamed Chowdhury, Ahmad Ghiaskar

**Affiliations:** Advanced Composites and Mechanics Laboratory, Department of Mechanical Engineering, Dalhousie University, Halifax, NS B3H 4R2, Canada; shahriarshadz@gmail.com (S.A.C.); ghiaskar@dal.ca (A.G.)

**Keywords:** basalt fiber, Recyclamine resin, epoxy, mechanical properties, sustainable composites, recyclable composites

## Abstract

The present study focuses on the mechanical performances of basalt fiber-reinforced composites based on the more environmentally friendly Recyclamine^®^ resin (BR) and conventional and widely used room-cured epoxy systems (BE). Specifically, the study probes the tensile and compressive responses of the composites fabricated by vacuum-assisted resin transfer molding. Experimental results revealed that the tensile strength of basalt–Recyclamine was higher than its counterpart (464 MPa compared to 390.9 MPa). At the same time, the BR performed only marginally better under compression, with a strength of 237.7 MPa compared to 233.9 MPa for BE. However, the BR demonstrated significantly enhanced ductility reflected by its greater compressive strain capacity (3.9% compared to only 1.1%). Different microscopic analyses unveiled distinct failure mechanisms, with more progressive failure patterns observed in BR compared with the brittle fracture characteristics of the BE composite. The performance of several micromechanical models was also investigated, with their results corroborating with the experimental results with varying degrees of accuracy. The statistical analysis showed great consistency in the results, with the CoV value below 10%. Experimental results indicated that the basalt–Recyclamine composites can be considered a promising sustainable alternative to traditional polymeric resin-based systems due to their balanced mechanical performance and environmental advantages.

## 1. Introduction

Fiber-reinforced plastic (FRP) composites have emerged in many application domains over the years. The superior mechanical properties of FRPs have expanded their applications in many facets of engineering, including automotive and aerospace, marine, infrastructure, and sporting goods [1]. Meanwhile, most current FRPs are based on non-recyclable thermoset resins, such as conventional epoxies, vinyl esters, and polyesters, which present significant environmental challenges at the end of their useful life [2]. Since the issue of persistent environmentally harmful materials and complex waste treatment regulations has become a challenging task for many industries, the demand for sustainable and recyclable composite materials that possess mechanical properties similar to those of traditional FRPs has gained significant attention, thus attracting the attention of many researchers. A good example of this increasing trend toward sustainability is the European Union’s ELV End of Life Vehicles directive, which mandates that all vehicle materials should be 85% recoverable [3,4,5]. Consequently, interest in developing recyclable thermoset resins that can deliver an environmental advantage without compromising the required mechanical properties for engineering applications has increased significantly in recent years. Fully “recyclable” in this context means that the cured thermoset composite can be subjected to chemical solvolysis treatment such that the fiber reinforcement and resin matrix can be reclaimed and recycled back to reusable forms.

When new recyclable composite systems are developed for engineering applications, estimating their mechanical properties, particularly their tensile and compressive properties, becomes essential. These properties are critical for evaluating the structural bearing loads as well as structural resilience across a wide spectrum of service conditions [6,7]. Tensile properties aid the designer in achieving the required stiffness of a given structural component, whereas the compressive properties govern the compressive properties and the buckling responses of the structure [8]. These properties have been of interest when new types of matrix materials, such as Recyclamine^®^, are introduced [9]. Recyclable thermoset matrices are generally classified based on reprocessing mechanisms, including reversible covalent bond networks such as Diels–Alder chemistry, dynamic covalent bonds, and cleavable linkers. Systems based on Recyclamine^®^ utilize cleavable amine–epoxy bonds that can be selectively broken through low-energy solvolysis under mild chemical conditions. This results in a thermoplastic matrix that can be recovered and reprocessed, closing the recycling loop while maintaining high mechanical integrity [10]. It should be noted that the chemical aspects of the resin are not within the scope of this work. Recyclamine is one of the most effective types of recyclable thermoset resin systems. Therefore, the knowledge of the performance of the compressive and tensile responses of composites made by such resins is of paramount importance because the current database for such information is significantly scarce. The scarcity of the database becomes even more critical when natural and sustainable fibers, such as basalt fibers that offer a competitive response to the commonly used fibers, are considered. This information is necessary to validate such composites for sustainable industrial scenarios [11]. Basalt fibers are obtained from natural volcanic rocks, with the promise of being a viable alternative to conventional reinforcement fibers such as glass and carbon. Basalt fibers offer good mechanical properties and resistance against chemicals and are environmentally friendly [12,13]. Moreover, recent developments, such as the creation of thermoset recyclable resins like Recyclamine^®^, allow new frontiers in the field for fully recyclable composite system designing with no loss of its structural performances [14]. Therefore, the combination of recyclable resins and sustainable fibers has received public attention.

Many researchers have conducted extensive work with sustainable and recyclable composite systems in recent years. Barczewski et al. [15] discussed Polyurethane (PUR) foam waste management through its use as a functional filler for basalt and epoxy composite hybridization. Das et al. [16] discussed the impact of the durability of flax fiber hybridization in composites against an off-the-shelf recyclable polymer matrix system for use in a large structural system. They considered the long-term performance of some UD flax FRPCs subjected to accelerated weathering and compared their results against the performance of some conventional composite materials [17]. Cicala et al. [18] synthesized various bioepoxy-based monomers, and totally recyclable bioepoxy resin hardened with recyclable amine. Cho et al. [19] conducted a deep investigation into the performance of flax fiber composites and concluded that a continuous flax fiber composite performed better than an equivalent glass/epoxy composite. Liu et al. [20] investigated the performance of bio-based epoxy resin derived from renewable sources against a comparable petroleum-based counterpart. Chilali et al. [21] evaluated the moisture absorption properties of flax–epoxy and flax–Elium^®^ composites. Yaghoobi and Taheri [22] examined the performance of composites consisting of a cross-ply basalt fabric and a recyclable thermoplastic resin, Elium^®^. Their study showed a 23.9% increase in the tensile strength of their basalt–Elium^®^ compared with an equivalent basalt–epoxy. In a recent work, Llanos et al. [23] studied the low and high-velocity behaviours of basalt–epoxy and basalt–Elium^®^ composites, presenting an in-depth overview of the dynamic response of the composites.

As stated, basalt composites have attracted significant attention lately. Another notable study is by Plappert et al. [24], who considered the performance of a unidirectional basalt–epoxy composite and reported a tensile modulus of 44.3 GPa with a strength of 1310 MPa. Amuthakkannan et al. [25] studied the hybrid composites of basalt and jute and established the optimal fiber volume ratio. Yasmeem et al. [26] studied Izod impact and Brinell hardness responses of continuous basalt fiber composites and showed that the composites exhibited nearly 100% superior impact strength compared to their comparable glass composite. Manikandan et al. [27] studied the effect of surface treatment of basalt fibers and found that the tensile strength of acid-treated basalt composite (246 MPa) was 77% higher than that of the untreated basalt fiber composite.

The above studies reveal that basalt fiber composites present enormous potential for use in various industrial applications. However, as stated earlier, the investigation of the performance of basalt fiber composites formed by recyclable thermoset matrices, like Recyclamine^®^, is relatively scarce. A considerable number of studies have concerned the mechanical properties of basalt fiber composites manufactured with conventional epoxy resins [28,29,30]. In contrast, a limited number of works have investigated recyclable thermoplastic matrices such as Elium^®^, and even a lesser number of works have considered basalt fiber composites using recyclable thermoset resins [23]. Several studies have explored the performance of Recyclamine^®^ technology, such as those by Dattilo et al. [31] and Ferrari et al. [32], which focused on chemical features and the life cycle analysis (LCA) rather than mechanical properties. In other words, the mechanical response of basalt–Recyclamine^®^ composites deserves further investigation.

This paper aims to characterize the tensile and compressive responses of basalt fiber-reinforced composites manufactured with the recyclable resin Recyclamine^®^ and compares their performance to conventionally made basalt–epoxy composites. The study’s main objective is to introduce a new recyclable, sustainable, and environmentally friendly composite and its potential as a viable composite for industrial applications. Moreover, the expected accuracy of the results produced by various micromechanical models, when applied to non-aerospace type composites, such as those used in this study, including the infrequently used but useful BACK-out Factor, is also the secondary objective of the present study.

This paper, therefore, will establish tensile and compressive strengths, elastic moduli, and failure mechanisms by tests conducted according to pertinent ASTM standards. Further discussion will be had on the microstructural characteristics and the failure modes using SEM analysis. Several micromechanical models, such as the Rule of Mixtures and Halpin–Tsai approaches, will also be applied to validate the experimental results. The concept of the Back-out Factor approach and its validity and accuracy in predicting compressive strength are also investigated. The preliminary evaluation presented in this paper is the initial basis for the continual efforts in establishing the other properties of such composites because, in many structural applications (e.g., in automotive and aerospace industries), the material encounters complex loading states, and the structural systems are expected to maintain its stability throughout their entire service life [23,33,34]. The work also establishes, for the first time, the correlation between the microstructural characteristics and mechanical properties, hence providing useful insights for future incorporation in the examination of sustainable and recyclable composite materials.

In contrast to the earlier work that examined either the mechanical or environmental response of recyclable composites in isolation, the current study is distinct in that it presents a holistic experimental evaluation of basalt fiber-reinforced composites that were processed using a recyclable Recyclamine^®^ resin. The current work methodically reports the tensile and compressive response of the recyclable composite and counterpart composite processed with a commonly used room-cured epoxy resin, following pertinent ASTM standard test guidelines. The study also relates the observed mechanical response to microstructural failure mechanisms via comprehensive SEM and optical studies. Additionally, the results from traditional micromechanical models are contrasted with experimental results to highlight their shortcomings when applied to non-aerospace-type composites. Furthermore, this study presents the first mechanical, micromechanical, and microstructural examination of basalt–Recyclamine^®^ composites, complementing the knowledge gap on green fiber-reinforced composites in the existing literature. The findings of this study pave the way for the creation of cost-effective, sustainable composite materials for structural applications in automotive, aerospace, and renewable energy systems in the future.

## 2. Materials and Methods

### 2.1. Materials

This study has explored the performance of two kinds of composite systems: basalt-reinforced Recyclamine (BR) and basalt-reinforced epoxy (BE). These materials were chosen to directly compare standard and recyclable composite systems while keeping the reinforcement properties similar.

**Figure 1:**
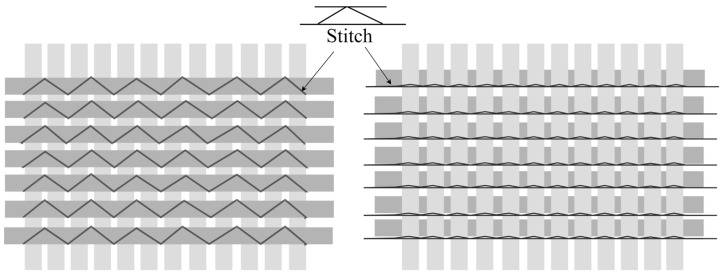
Schematic representation of the stitches used in bidirectional fabrics.

The reinforcement material was a 669 g/m^2^ stitched cross-ply basalt fiber fabric developed by Zhejiang GBF Basalt Fibre Co., Ltd. (Dongyang, China) and acquired from Advanced Filament Technologies LLC (Houston, TX, USA). The fibers were made with 13-micron roving and had a density of 2.75 g/cm^3^ [27,35]. The mechanical features of the basalt fibers included a tensile strength of 4840 MPa, a tensile modulus of 89 GPa, and an elongation at break of 3.15%. The choice of the biaxial stitched fabric over woven types was due to its better fatigue resistance and more even fiber distribution because the non-crimp nature of stitched fabrics removes the fiber waviness often seen in woven structures. Figure 1 shows a schematic representation of the stitched biaxial fabric.

**Figure 2:**
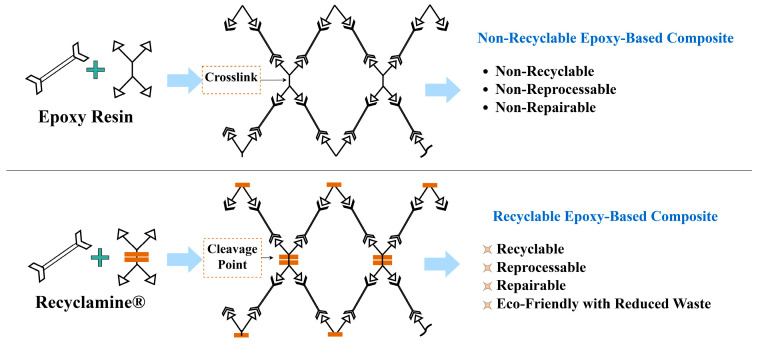
Schematic comparison of polymer links in conventional epoxy and Recyclamine^®^ systems.

For the matrix systems, the first material used was the Briozen Recyclable Series, utilizing Recyclamine^®^ Technology (Epotec YDL5557 THR9357), provided by Aditya Birla Chemicals (Bangkok, Thailand) Ltd. This new two-component system is a big step forward in recyclable thermoset technology. The Recyclamine^®^-based thermoset matrix contains cleavable amine–epoxy linkages, which are prone to solvolysis under low-energy conditions and thus easily re-processable into a reusable thermoplastic form. This facilitates material recoverability and re-processability and, hence, promotes green composite manufacturing [36]. Figure 2 schematically compares the polymer network structures of conventional thermoset epoxies vs. Recyclamine^®^ at cleavable linkage points to achieve recyclability.

Recyclamine^®^ resin system facilitates the recovery of fiber and matrix components via a low-energy solvolysis process, thus enhancing recyclability in fiber-reinforced composites. Due to its lower reactivity and lower heat generation compared to standard epoxies, it is compatible with different processing environments. Its relatively lower viscosity also offers high fiber wet-out efficiency, thus making it suitable for infusion processing [37]. The main physio-mechanical properties, mixing ratio, and curing conditions recommended by the manufacturer for this study are given in Table 1.

The second matrix used was the room-cured West System^®^ epoxy, consisting of 105 resin and 206 hardener. This widely used conventional epoxy system served as a benchmark for comparison of mechanical properties. The system had a viscosity of 725 mPa.s and a working time of 90–110 min, with full cure at room temperature taking 10–15 h. The mechanical properties of neat, cured epoxy are 50 MPa tensile strength, 3.172 GPa tensile modulus, and 4.5% elongation at break [38]. The key physico-mechanical properties of the neat, cured West System^®^ 105/206 epoxy system are summarized in Table 2. Both matrix systems were chosen to make sure of good fiber impregnation and fit with the vacuum-assisted resin transfer molding (VARTM) process used in this study.

### 2.2. Composite Fabrication

All composite panels were made using the vacuum-assisted resin infusion molding (VARTM) process. Before making the panels, a 10 mm thick flat aluminum plate was cleaned with acetone, and a release agent was applied to help in panel removal. The fabric preforms were cut to the needed dimensions, keeping the 0/90° fiber direction. The stacking order was [0/90]_ns_, where ‘n’ was set based on how thick the test specimens should be. Table 3 shows the ASTM suggested specifications for the panels meant for different mechanical tests, including dimensions and the number of fiber layers. Each specimen group consisted of six specimens.

The VARTM fabrication was assisted under a 1-bar vacuum in a sealed mold to take out trapped air and consolidate the fiber preforms. The resin systems were mixed in their ratios (100:27 for Recyclamine^®^ and 5:1 for epoxy), as specified by the resin developers. They were then degassed in a vacuum chamber to eliminate air bubbles. A fiber:resin ratio of 1:1.2 was used to reach the desired fiber:volume ratio and account for resin loss during processing [39]. The resin was infused under vacuum pressure to ensure even flow through the spiral tubes and distribution media. The resin was kept at around 24 °C throughout infusion to obtain the optimum viscosity and flowability. The injection was carried out at a controlled flow rate of around 60 g/min, keeping the mold temperature at ambient (~24 °C).

**Figure 3:**
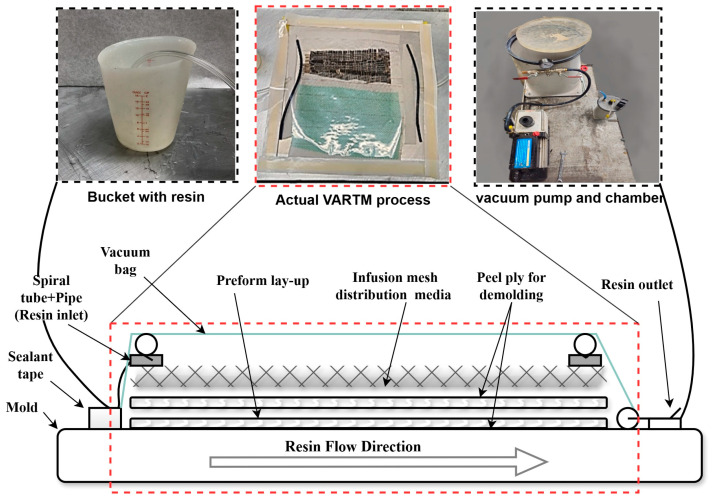
Schematic diagram of the VARTM process and photographs of the actual manufacturing setup.

The curing processes were different for the two resin types. The West System^®^ epoxy panels were cured at room temperature for 24 h. The Recyclamine^®^ panels went through two stages: a curing cycle of 24 h at room temperature, followed by a post-cure cycle at 80 °C for 8 h in a digitally controlled oven. As stated earlier, these curing procedures were selected based on the manufacturers’ specifications to ensure optimal material performance. Figure 3 shows a schematic of the VARTM process along with pictures of the manufacturing setup.

### 2.3. Test Equipment and Instrumentation

Mechanical testing was performed on an MTS digital servo-hydraulic universal testing machine. The strain was measured by using 350-ohm strain gauges (Micro-Measurements, Wendell, NC, USA) and a laser extensometer (Model LE-05, Electronic Instrument Research, PA, USA). Data acquisition involved a Compact DAQ chassis 9172 and an NI 9237 module to capture the strain, with RJ 50 cables for signal transmission.

Strain gauges were mounted in a quarter-bridge configuration with the assistance of NI 9945 quarter-bridge completion modules. The acquisition was carried out with NI Signal Express 2015 software for real-time recording and display. The laser extensometer provided a non-contacting measurement of strain during tests by measuring the displacement of the gauge length within the two reflective tapes.

The calibration and verification of the measurement systems were performed using aluminum alloy 3003 as a control material. The strain gauges gave reproducible results with an average elastic modulus of 65.2 GPa, while the laser extensometer gave slightly more scatter at 65.2 ± 5 GPa. Both values were comparable to the standard aluminum modulus, 68.9 GPa. This dual measurement approach ensured the accuracy of the measured strains.

### 2.4. Tensile Testing

**Figure 4:**
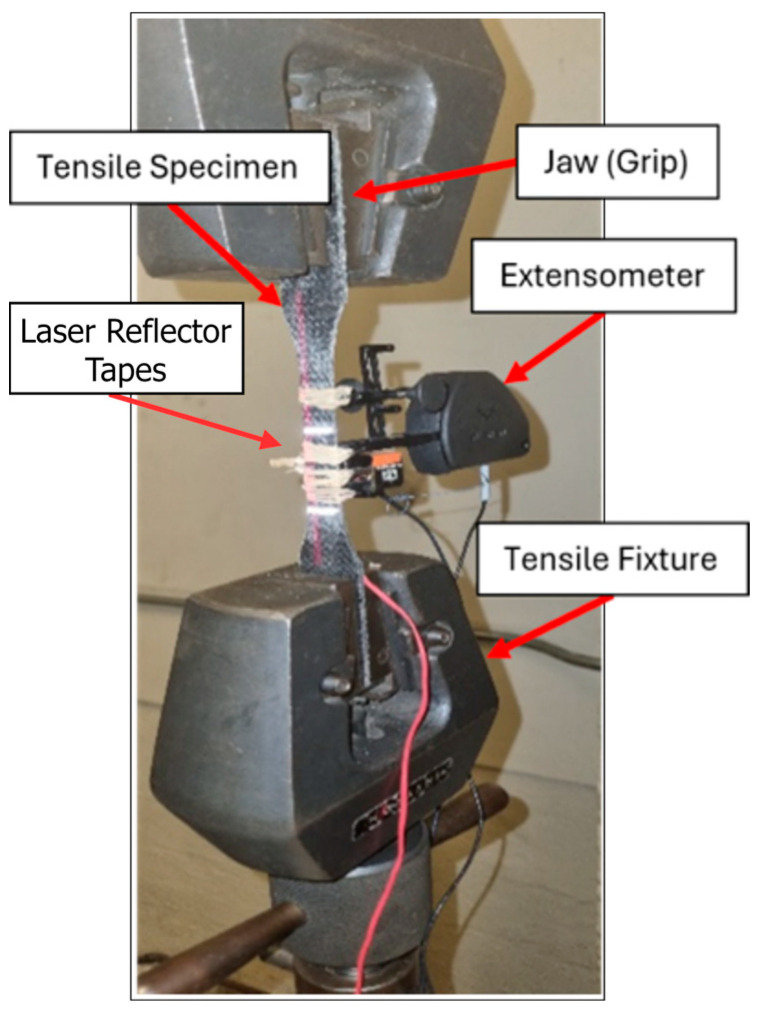
Basalt–epoxy specimen during tensile testing.

Tensile tests were carried out on an MTS universal test machine [40]. Dog-bone test specimens with dimensions of 250 × 25 (at the gauge length) × 2.5 mm^3^ were used for both composites. Waterjet cutting was used as per the CAM design to realize dimensional accuracy and precision without affecting the materials’ properties. Figure 4 depicts the test setup of basalt–epoxy specimens used in tensile testing.

The test configuration utilized both systems for strain measurement: bonded strain gauges and a laser extensometer. Strain gauges were bonded at the specimen gauge length, and reflective tape markers were also placed within the gauge length for strain measurements using the laser extensometer. The tests were conducted at a nominal rate of 5 mm/min according to ASTM D638, such that the tests would be completed within 5 min.

Data collection was performed at 2048 Hz through both the MTS software and the NIDAQ system. Data collected through the MTS included measured force (kN) and actuator displacement (mm) as a function of time. The tensile stress (σ), strain (ε), and elastic modulus (E) were calculated from the data collected through the strain reading using the basic mechanics of materials relationships.

### 2.5. Compression Testing

Compression tests were performed in accordance with the ASTM D6641M-16e2 standard on a Combined Loading Compression (CLC) test fixture [41]. Rectangular specimens with dimensions of 140 × 25 × 4 mm^3^ were cut, of which the thickness was determined using the standard Euler buckling prevention equation:

(1) $$h\geq \frac{l_{g}}{0.9069\sqrt{\left(1-\frac{1.2F^{cu}}{G_{xz}}\right)\left(\frac{E^{c}}{F^{cu}}\right)}}$$

where h is specimen thickness, $l_{g}$ is, gauge length, $F^{cu}$, $E^{c},$ and $G_{xz}$ are the expected ultimate compressive strength, flexural modulus, and through-thickness shear modulus, respectively.

The CLC equipment had two sections, one section equipped with a pair of roller-bearing mechanisms to ensure concentric vertical motion. The specimen was held firmly in the equipment with the assistance of bolts tightened to a torque of 2.5 Nm. The two ends of the specimen were in contact with the top and bottom plates of the MTS. The strain was measured using both strain gauges and a laser extensometer, with strips of retroreflective tape applied parallel to the width of the specimen at designated gauge positions.

**Figure 5:**
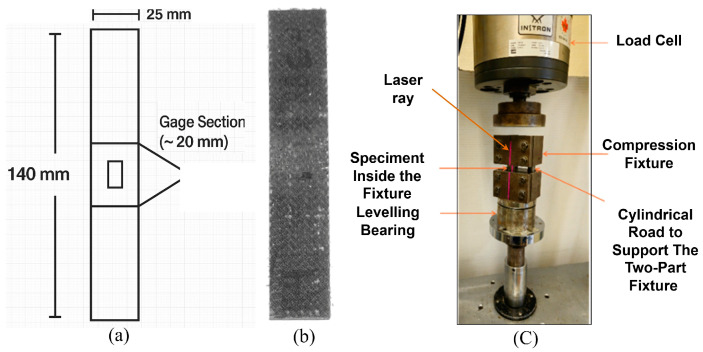
(**a**) Schematic of the compression test specimen, (**b**) an example of a basalt–Recyclamine sample, and (**c**) the experimental setup used for the compression test.

Testing was conducted at a 1.3 mm/min loading rate according to the standard. The MTS quantified load and displacement during the tests while the DAQ captured the strains. The testing was continued until visible failure was noted, which was normally followed by micro-buckling of the fibers, delamination, or shear crimping. Five specimens of each composite type were tested for statistical confidence. Post-failure analysis was performed through observation of the modes of failure and comparison of basalt–epoxy and basalt–Recyclamine samples. Compressive stress–strain behaviour was calculated using the same basic mechanics of materials equations akin to tensile testing. Figure 5 illustrates a typical compression specimen and the test setup.

### 2.6. Micromechanical Assessment of Tensile and Compressive Properties

Several micromechanical models were employed to predict the mechanical properties of the basalt fiber-reinforced composites and verify their predictive accuracy. The widely used Rule of Mixtures (RoM) was employed to predict composite elastic properties [42]. Perfect interfacial bonding between matrix and fiber and equal strains under the influence of applied load are some of the assumptions used in the development of RoM, mathematically represented as follows:

(2) $$E_{1}=E_{f}.V_{f\;}+E_{m}.V_{m\;}$$

where $E_{f}$ and $E_{m}$ are modules of elasticity for the fiber and matrix, respectively, and $V_{f\;}$ and $V_{m\;}$ are volume fractions of their respective materials.

The Halpin–Tsai model [43] was also employed to find other mechanical properties considering geometric aspects and orientation of fibers:

(3) $$M=\left(\frac{1+\xi \eta V_{f}}{1-\eta V_{f}}\right)M_{m}$$

where the coefficient η is equal to:

(4) $$\eta =\frac{\frac{M_{f}}{M_{m}}-1}{\frac{M_{f}}{M_{m}}+\;\xi }$$

In this case, M is utilized to represent the desired elastic properties (E1, E2), whereas $M_{f}$ and $M_{m}$ are utilized to represent the respective fiber and matrix values, respectively. Two (2) was the reinforcement factor, ξ, when the transverse modulus of elasticity of the composite material was being calculated.

The Back-Out Factor (BoF) approach was also utilized for establishing the tensile and compressive strength of unidirectional (UD) composites. BoF is computed by multiplying the experimentally obtained result from tensile and compressive tests on cross-ply laminate [44,45,46] to obtain the UD properties. The factor is the axial stiffness ratio of the cross-ply laminate to the unidirectional layers. For a symmetric and balanced cross-ply lamina with design [0/90]_ns_, the equation is:

(5) $$BoF=\frac{Q_{11}^{0}A_{22}-Q_{12}^{0}A_{12}t}{A_{11}A_{22}-A_{12}}$$

where $Q_{11}^{0}$ and $Q_{12}^{0}$ are the reduced stiffness matrix elements for a unidirectional ply and $A_{11}$, $A_{12}$, and $A_{22}$ are the axial stiffness terms for a unidirectional laminate in-plane stress.

In the absence of any test data for transverse modulus, Hart-Smith has presented a generalized equation based on the simplified rule of mixture in conjunction with thin plate theory [47]. This technique assumes the composite to be a thin plate where the effect of Poisson’s ratio can be ignored:

(6) $$\sigma_{11}=\frac{\sigma_{x}}{V_{f_{0}}+(1-V_{f_{0}})\;\frac{E_{2}}{E_{1}}}$$

where $V_{f_{0}}$ represents the volume fraction of fibers oriented at 0° in the cross-ply laminate, $\sigma_{x}$ denotes the tensile or compressive strength of the cross-ply composite, and $\sigma_{11}$ refers to the strength of the corresponding unidirectional (UD) composite.

Another approach is given by the Military Handbook (MIL-17-F), where the transverse modulus effects (E2) are negligible [47].

(7) $$\sigma_{1}=\frac{E_{1}}{E_{x}}\times \sigma_{x}$$

where $\sigma_{x}$ is the tensile/compressive strength of the cross-ply laminate, and $\sigma_{1}$ is the strength of its unidirectional laminate.

**Figure 6:**
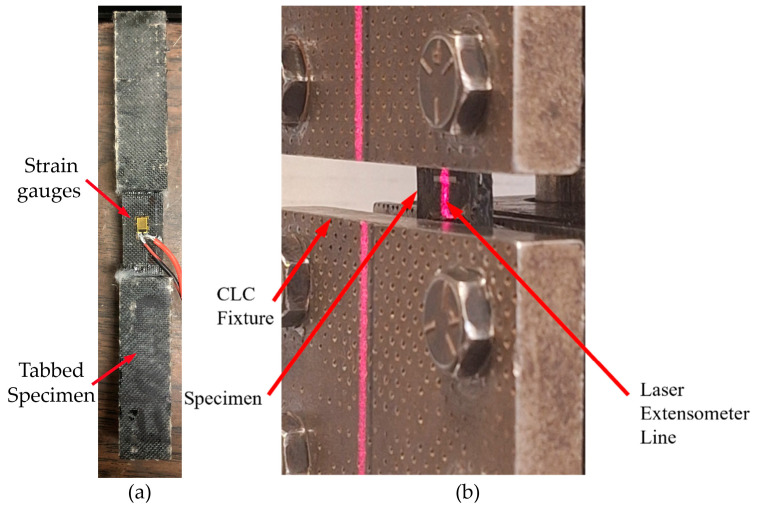
(**a**) Specimen with tabs applied, and (**b**) specimen positioned within the testing apparatus.

To validate the theoretical predictions, an attempt was made to convert the biaxial fabric to a uniaxial configuration by painstakingly removing the orthogonal fibers from the biaxial fabric. This attempt was made since, at the time, we could not source any unidirectional basalt fabric. The resulting fabric was not ideal as it was somewhat wavy, and the fiber distribution did not remain as uniform as desired. The fabric was used to generate compression test specimens with epoxy and Recyclamine matrices. The tests were carried out in accordance with the ASTM D6641M [41], using the Combined Loading Compression (CLC) fixture. To prevent Euler buckling and ensure that the loading was restricted to the gauge region, the specimens were tabbed with care using fiberglass. Strains were monitored with a laser extensometer, as shown in Figure 6.

These analyses were conducted to gain better insight into the material’s micromechanics and verify various prediction methods. Statistical analysis entailed calculating the average, standard deviation, and coefficient of variation for experimental and theoretical results.

### 2.7. SEM and Digital Microscopic Analyses

After tensile and compression testing, the basalt fiber-reinforced composites’ microstructure and failure mechanisms were analyzed using Scanning Electron Microscopy (SEM). The Hitachi S-4700 FE SEM (Hitachi High-Tech Inc., Etobicoke, ON, Canada) with magnifications up to 500,000× was utilized for the detailed images of the fiber–matrix interaction, fracture morphology, and internal structural irregularities. In the case of impacted specimens, regions near the failure zone were selected for detailed inspection. Small portions were precisely sectioned from these areas, coated, and mounted on SEM stubs for imaging.

The SEM showed major failure mechanisms (e.g., fiber breakage, matrix cracking, fiber pull-out, and delamination), revealing the composite’s behaviour under mechanical loads. The digital microscopic analysis technique was used to evaluate defects at a larger scale, such as delamination, cracks, and surface irregularities. The findings of the examination corroborate the SEM results, which will be shown later. When used in synchrony, these methods of analysis provided a deep and clear understanding of the acquired knowledge about the composite’s structural integrity and the distinct ways of failure it could face.

## 3. Results and Discussion

The mechanical properties and recyclability of basalt fiber-reinforced composites using Recyclamine^®^ resin were compared to those of conventional epoxy systems. The increasing environmental awareness of composite waste disposal and strict regulations has made the development of sustainable, recyclable composites with maintained mechanical performance a priority. This study will provide an important understanding of the feasibility of Recyclamine^®^ as an environmentally friendly alternative to conventional epoxy systems in structural applications.

The introduction of Recyclamine^®^ technology represents a significant development step toward sustainable and recyclable composite manufacturing. In contrast to the conventional thermoset matrices, which cannot be recycled, the cleavable chemical structure of Recyclamine^®^, under certain conditions, enables complete recyclability of the matrix and reinforcing fibers. This property and the natural sustainability of basalt fibers give rise to a composite system that will answer the performance–environmental needs.

### 3.1. Tensile Properties

**Figure 7:**
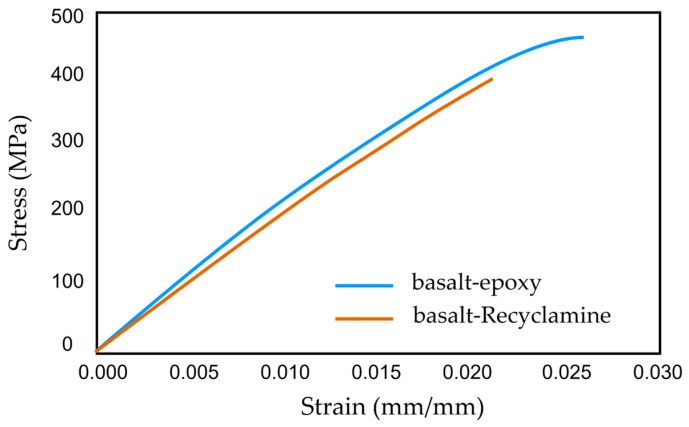
Comparison of the tensile stress–strain responses for basalt–epoxy and basalt–Recyclamine composites.

The basalt–epoxy composite, with an average tensile strength of 464 MPa, outperformed the basalt–Recyclamine’s 390.9 MPa. However, the basalt–Recyclamine exhibited a unique property—better ductility, with an ultimate strain of 1.9% compared to the 2.4% of the basalt–epoxy. The tensile modulus of elasticity was 20.2 GPa for the basalt–Recyclamine and 22.0 GPa for the basalt–epoxy, indicating a slightly stiffer response for the epoxy-based system. The stress–strain behaviour of both composites, as shown in Figure 7, highlighted the intriguing ductility of the basalt–Recyclamine composite. Whereas the basalt–Recyclamine composite’s tensile strength was lower than that of the basalt–epoxy system, its mechanical performance renders it suitable for lightweight structural applications where sustainability is of paramount concern.

The statistical analysis revealed consistent and repeatable results for the test specimens. The coefficients of variation of 5.6% and 7.9% for the tensile strength of basalt–epoxy and basalt–Recyclamine composites, respectively, reflected the reliability of the results. The values of the calculated modulus values from the data were also consistent, with coefficients of variation of 8.7% and 4.1% for basalt–epoxy and basalt–Recyclamine, respectively. These results, presented in Table 4, provide a reliable and comparative assessment of the tensile properties in detail.

**Figure 8:**
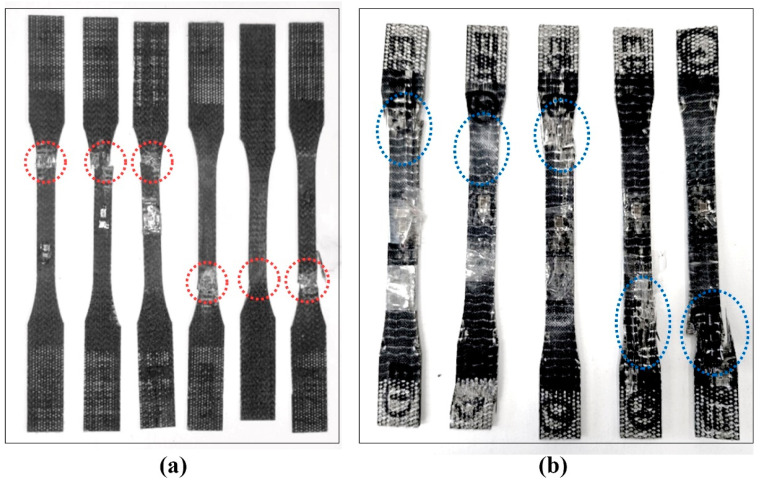
Fracture locations (identified by dotted red and blue boundaries) of tensile specimens after testing: (**a**) basalt–epoxy composite, (**b**) basalt–Recyclamine composite.

Failure locations in the tested specimens are indicated in Figure 8, showing typical failure modes. The failure in basalt–epoxy specimens (Figure 8a) was near the grip region (red circle) with a neat, brittle fracture surface and minimal fiber pullout. This failure mode is indicative of the strong but brittle nature of the basalt–epoxy composite. On the other hand, the failure region in the basalt–Recyclamine specimens (Figure 8b) exhibited greater fiber pullout and delamination, indicative of a more progressive failure mode. This mode of failure suggests the more ductile nature of the basalt–Recyclamine composite. These failure modes concur with the stress–strain behaviour observed during testing, where the basalt–Recyclamine composite showed greater ductility compared to the brittle failure of basalt–epoxy samples.

Most of the failures occurred in the region of the grip area rather than the gage length center of the specimen, primarily due to stress concentration effects around the grips. Such failure is, however, acceptable according to the ASTM D638 standard. The surface quality of the specimens was also somewhat varied, with basalt–Recyclamine having superior-quality surfaces compared to the relatively rougher surfaces of basalt–epoxy specimens due to the lower viscosity of the Recyclamine resin during processing. This difference in surface quality could have implications for the application of these composites, with the smoother surface of the basalt–Recyclamine potentially offering better adhesion in certain applications.

### 3.2. Compressive Properties

**Figure 9:**
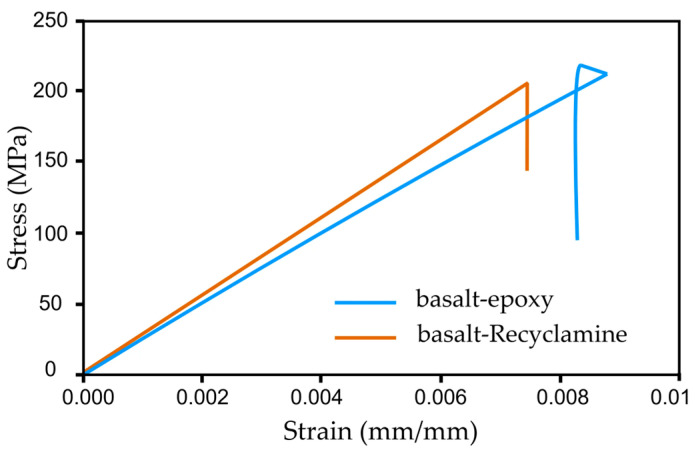
Comparison of the stress–strain comparison between basalt–epoxy and basalt–Recyclamine composites.

The compressive response of basalt–Recyclamine composite was superior, with an average ultimate compressive strength averaging 237.7 MPa for basalt–Recyclamine, slightly outperforming the 233.9 MPa observed in the basalt–epoxy counterpart. The readings of strain indicated a much greater ultimate strain of 3.9% for basalt–Recyclamine than the 1.1% for basalt–epoxy, indicating much more ductile compression behaviour. The compressive modulus of elasticity was also higher in basalt–Recyclamine at 27.5 GPa, than in basalt–epoxy at 24.0 GPa. The typical compressive stress–strain curves for the two composites are presented in Figure 9.

Statistical analysis indicated good repeatability among test samples with coefficients of variation of 6.9% and 6.3% for compressive strength in basalt–epoxy and basalt–Recyclamine composites, respectively. Modulus values proved to have very good repeatability with coefficients of variation of 2.5% for basalt–epoxy and 5.6% for basalt–Recyclamine. The above results are presented in Table 5, which provides a general comparison between compressive properties.

**Figure 10:**
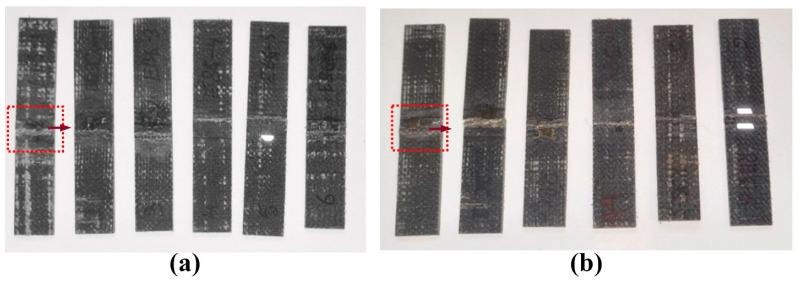
Similar fracture locations identified by dotted red boundaries in compression-tested specimens after failure: (**a**) basalt–epoxy, (**b**) basalt–Recyclamine.

Post-failure analysis of the test samples in Figure 10 indicates variation in failure modes of the two systems. Basalt–epoxy samples showed micro-buckling and lesser fiber pullout, indicating stronger fiber–matrix bonding but more brittle failure. Basalt–Recyclamine samples showed greater fiber pullout, reflecting comparatively lower fiber–matrix bonding but more ductile failure with greater energy absorption. The relatively more ductile Recyclamine matrix appeared to support greater fiber deformation and transfer loads from one fiber to another more effectively.

The relatively lower compressive than tensile properties for both composites, which is commonly experienced, can be attributed to the nature of the compression test and the shorter gauge length of specimens, leading to Saint-Venant’s stress effect [48]. Also, void content in a given composite affects mechanical properties of composites, more so under compression than tensile state, by inhibiting efficient load transfer between fibers and promoting initiation of microcracks.

### 3.3. Comparative Analysis of Mechanical Properties

**Figure 11:**
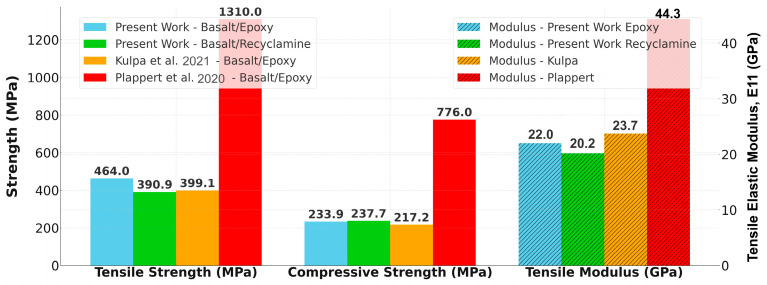
Comparison of tensile strength, compressive strength, and tensile modulus for various basalt fiber composite systems [24,49].

A comparative analysis of mechanical properties obtained in the current research and available literature is presented in Figure 11. The figure illustrates the comparison between tensile and compressive strengths, and the tensile modulus of various basalt fiber composite systems investigated by several researchers.

The BE investigated in this study exhibited the highest tensile strength (464.0 MPa) among all other conventionally processed composites, which was also higher than that of the BR (390.9 MPa) and the BE investigated by Kulpa et al. (399.1 MPa) [49]. The much higher tensile strength of the BE of Plappert et al. [24] (1310.0 MPa) is attributed to their use of prepreg, and autoclave processing tends to generate better consolidation, leading to stronger fiber–matrix interfaces and greater fiber volume fractions.

The compressive strength data revealed an intriguing pattern: the BR composite’s strength (237.7 MPa) was slightly higher than our BE’s strength (233.9 MPa, and also higher than Kulpa et al.’s BE (217.2 MPa). Again, Plappert et al.’s autoclave-processed materials were far superior in compressive strength (776.0 MPa), clearly illustrating the predominant influence of production processes on mechanical properties.

The repeatability of the experimental results can be observed through the coefficient of variation (CV) values shown in Table 4 and Table 5. For tensile properties (Table 4), two composites had good repeatability with CV values of 5.6% and 7.9% for strength and 8.7% and 4.1% for elastic modulus for both BE and BR systems, respectively. Likewise, the compressive strength results presented in Table 5 also showed good consistency with CV values of 6.9% for BE and 6.3% for BR. Nonetheless, a large variation of 29.2% can be seen in the compressive strain results of BR, which is attributed to the more complex stress state within the very small gauge length of the specimens, which can be significantly influenced by the existence of nonuniformly distributed voids in the composite. It is noted that the significant coefficient of variation (29.2%) of the compressive strain of basalt–Recyclamine specimens indicates a high degree of data scatter in addition to describing the complexity of the stress state. The scatter is attributed to the non-uniform void distribution, resin-rich zones, and non-uniform fiber distribution. Replicating compression testing with the increased number of specimens will be a part of our future work to examine the influence of such parameters with a greater statistical certainty.

The concurrence of the results obtained from our composites and those of Kulpa et al. [49] is attributed to the use of a similar vacuum-assisted process. Moreover, although the basalt–Recyclamine composite exhibited relatively lower tensile properties compared to the conventional basalt–epoxy systems, its similar compressive properties and enhanced ductility, and its useful recyclability attribute, render it a viable, environmentally sustainable alternative. The disparity in properties between our vacuum-assisted hand layup processed composites and Plappert et al. [24] autoclave-processed composites unequivocally demonstrates the significant effect of manufacturing processes on a composite’s mechanical properties. Nonetheless, and most importantly, the ability of a fully recyclable matrix such as Recyclamine and sustainable natural fibers such as basalt fibers places basalt–Recyclamine composites at the forefront in material selection for fabricating structural composites, where a balance between mechanical performance and environmental friendliness is desired. This offers a viable and cost-effective solution for various industries to meet the increasingly stringent environmental regulations without.

### 3.4. Micromechanical Analyses

#### 3.4.1. Rationale

The main objective of comparing the micromechanical model in this work is twofold. First, it is attempted to illustrate the important outcome of when one applies the available micromechanical models to the types of composites investigated in this study. It should be noted that the development of the available models was essentially based at the request and on the support of the aerospace industry. Moreover, the models were validated against the test results obtained from aerospace-grade (i.e., non-prepreg and non-autoclaved composites). Those composites have significantly more uniform, well-distributed, and non-contiguous fiber distribution, with essentially no void due to autoclave processing. Such composites are substantially different from the composites used in most non-aerospace applications. Therefore, one should expect various degrees of discrepancies in the results obtained by the models when applied to non-aerospace type composites.

It is also paramount to understand the reason for the inclusion of the Back-out Factor in our study. A simple search of the literature reveals a large number of scholarly articles devoted to examining the compression test of composites and the development of various ASTM test guidelines due to the inherent challenges encountered in such a test. As part of combating the challenge, researchers have developed various ASTM-approved fixtures to minimize buckling and slippage of composite specimens (e.g., these include the Celanese and IITRI fixtures (both following ASTM D3410 [50]), the Boeing/SACMA fixture (ASTM D6484 [51]), the CLC fixture (ASTM D6641 [41]), and the modified ASTM D695 [52] Compression Test Fixture (the same as Boeing BSS 7260 [53]).

Specimen grip-induced stresses developed in both tensile and compressive testing promote end crushing in uniaxial testing, necessitating the use of tabs, which increases the time, effort, and materials required. Cross-ply laminates were used in our study to eliminate the need for tabs. However, extracting a reliable strength value from cross-ply specimens remains a challenge.

The Back-out Factor was developed specifically to improve strength predictions for untabbed, cross-ply composites, and its development and validation were based on aerospace-grade materials [54]. It was shown that the Back-out Factor could yield relatively accurate values for untabbed cross-ply specimens compared to the use of traditional ROM and modified micromechanical models. Therefore, this method was also selected in this study to investigate its applicability.

#### 3.4.2. Results

The theoretical predictions of various micromechanical models are compared to experimental results for both composite systems. In the case of basalt–epoxy composites [24], for example, RoM provided an estimate for E_1_ as E_11_ of 43.8 GPa, while the estimate for E_2_ by the Halpin–Tsai model was at 11.9 GPa. These predictions, summarized in Table 6, compare well with values from literature reports on similar basalt–epoxy systems [13,54,55].

The tensile strength values predicted using the different approaches ranged from 703.1 MPa obtained using the BoF approach to a maximum of 1002.2 MPa obtained with the Simplified RoM and CLPT method. The Military Handbook alternative approach obtained an intermediate value of 923.9 MPa. The predicted compressive strength values ranged between 354.5 MPa (BoF) and 504.9 MPa, predicted by the Simplified RoM and CLPT methods, with an intermediate value of 427.1 MPa obtained using the Military Handbook alternative approach.

Similar comparison for basalt–Recyclamine composites, presented in Table 7, yielded RoM longitudinal modulus estimation of 41.4 GPa and Halpin–Tsai transverse modulus estimation of 12.2 GPa. The BoF approach provided compressive strength estimation of 349.8 MPa, while Simplified RoM and CLPT estimated 494.5 MPa and the Military Handbook approach gave 357.4 MPa estimation.

As described earlier, the method of conversion of the biaxial fabric to unidirectional fabric did not result in a desirable, uniformly dispersed, and contiguous fashion with straight fibers. Therefore, an effort was made to acquire manufactured unidirectional fabric. This attempt was made to further verify the integrity of the theoretical predictions. The compression specimens extracted from the composites made from the unidirectional fabric were tested in comparison. The results of these tests are also compared in Table 6 and Table 7, wherein the experimental compressive modulus and strength values are juxtaposed with the values derived from micromechanical models. In the basalt–epoxy composite, the experimental compressive modulus was 30.42 GPa and compressive strength was 254.9 MPa, whereas for basalt–Recyclamine, the experimental modulus was 29.23 GPa and strength was 274.9 MPa. The experimental modulus in both cases compares reasonably well with the analytical predictions, but the experimentally derived compressive strengths for the two systems were far below those obtained from theoretical calculations. Please also note the improvement in the COV values of the uniaxial specimens’ test results against the large COV associated with the cross-ply specimens. In fairness, the large COV of cross-ply specimens is partially attributed to the potential non-parallel far edges of the specimens that were in contact with the loading and support plates. It can be appreciated that a minor deviation can significantly influence the distribution of the desired uniform stress within the gauge length of the tested specimens.

**Figure 12:**
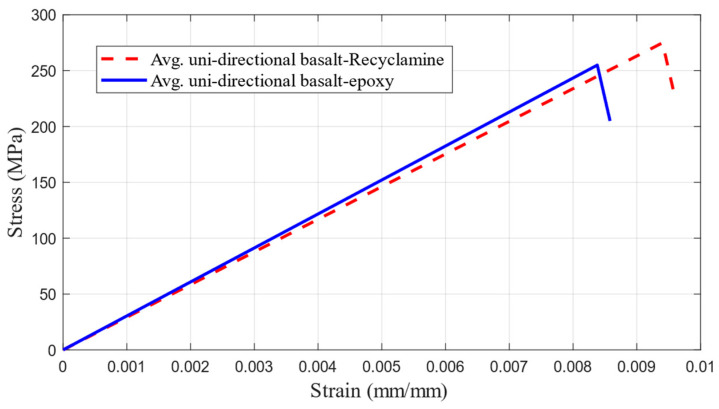
Average compressive stress–strain curve of basalt–epoxy composite and basalt–Recyclamine basalt.

Figure 12 illustrates the average compressive stress–strain curves for the unidirectional test specimens of the two matrix systems. As can be seen, both curves exhibited almost linear elastic behaviour to failure, although differences in stiffness and maximum stress between the two systems can be seen.

**Figure 13:**
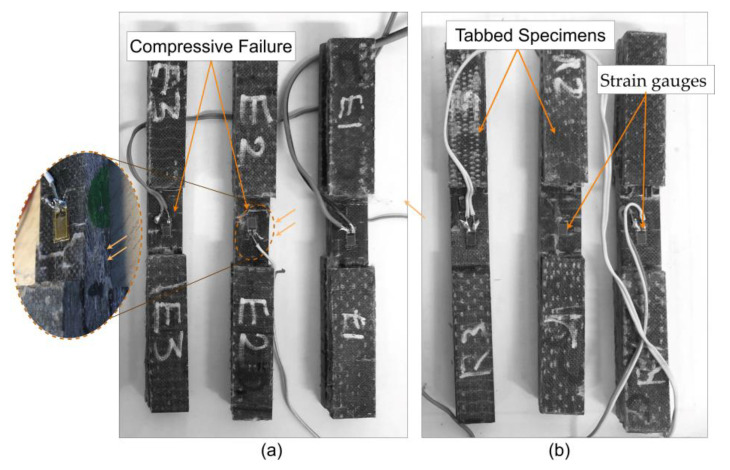
Post-compression test images of unidirectional basalt fiber composite specimens: (**a**) basalt–epoxy; (**b**) basalt–Recyclamine.

In general, the theoretical models’ predictions of the elastic moduli were found to be conservative, whereas the predicted strength values were nonconservative in most cases. This can be attributed to several factors, including, but not limited to, the presence of voids, fiber misalignment, and the assumptions held in the theoretical models, which assume an ideal condition that cannot be achieved using the vacuum-assisted hand layup fabrication method. Previous studies showed that such models were more accurate for property predictions for aerospace-grade composites manufactured under optimized conditions. Nevertheless, the comparison of experimental and theoretical values provides an important insight into how these models’ predictions should be treated when applied to non-autoclaved, non-prepreg reinforced composites. Nevertheless, these micromechanical models ought to be considered as approximate guidelines because of the large deviations noted, especially when predicting strength. Extension of these micromechanical models to structural design details in composites must be accomplished cautiously and with experimental calibration and validation, particularly in composites manufactured by VARTM or any other non-optimum process. Figure 13 shows the representative compressive failure images of unidirectional basalt–epoxy and basalt–Recyclamine test specimens. As seen, definite signs of compressive failure are detected in the test specimens, and the use of tabs and strain gauges can also be noted, which provided proper load introduction and accurate measurement of the strains during tests.

### 3.5. Microscopic Analysis

As briefly stated earlier, the microstructural failure mechanism and behaviour for both composite systems were analyzed through SEM and Digital Microscopy.

**Figure 14:**
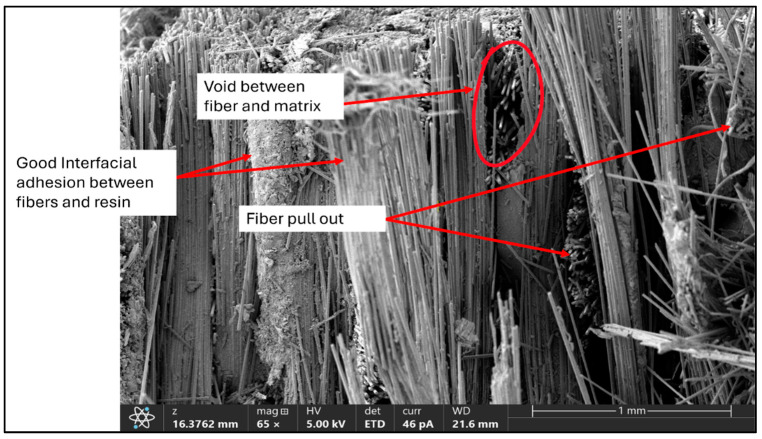
SEM image (×65) of the fracture surface of a basalt–epoxy composite specimen, showing good fiber–matrix adhesion, localized voids, and fiber pull-out regions.

**Figure 15:**
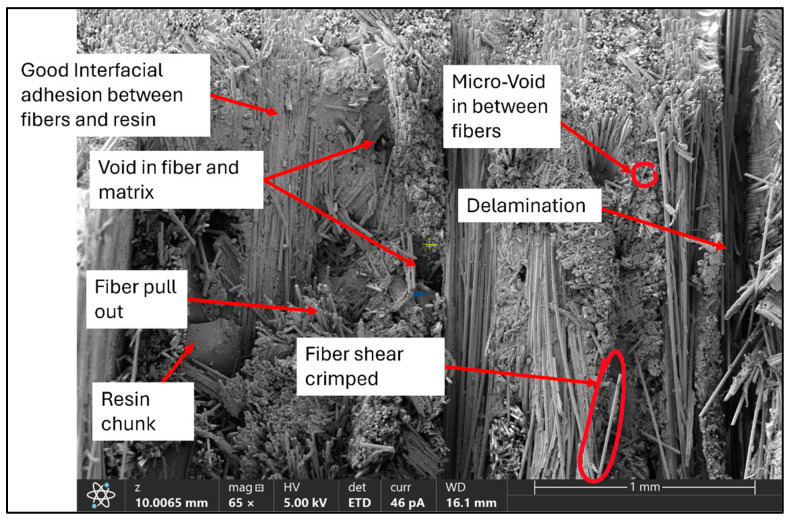
SEM image (×65) of the fracture surface of a basalt–Recyclamine composite specimen, highlighting delamination, fiber shear crimping, micro-voids, and resin chunks.

The SEM micrographs illustrated in Figure 14 and Figure 15 reveal relevant information about failure behaviour and adhesion in both composites at interfaces. Although full vacuum-assisted infusion and stitched fabric reinforcement were used to promote uniform resin distribution, some localized fiber clusters were observed, which is inherent to somewhat loosely stitched fabric architectures. For BE samples, high adhesion at interfaces between fibers and matrix is evident, with the presence of a few voids potentially acting as sources for stress concentrations. For BR samples, a similar intensity of interface quality is observed but accompanied by a clear presence of fiber pull-outs, which indicates variation in the progression of the failure mechanism.

**Figure 16:**
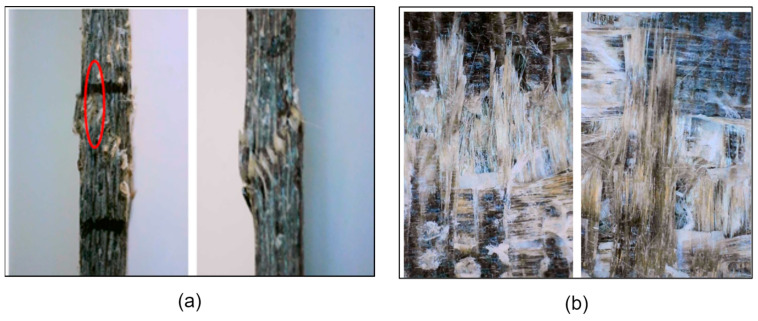
Microscopic images of failure modes under (**a**) tensile, (**b**) compressive loading states.

Analysis through digital microscopy conveyed deeper information about failure behaviour under tension and compression. The micrographs in Figure 16 illustrate failure behaviour in both composites under tension and compression loading states. Under tension loading (see Figure 16a), BE samples showed high breakage of basalt fibers with negligible evidence of fiber pull-out, signifying good fiber–matrix adhesion. For BR specimens, high fiber pull-out and delamination are observed, signifying a relatively progressive failure mechanism.

Under compressive loading (see Figure 16b), the BE composite showed micro-buckling and shear-crimping, accompanied by delamination in its intermediate plane. For basalt–Recyclamine samples, localized distribution of damages with filament crushing and breakage in its matrix, in agreement with its high strain capacity in tests in terms of mechanical properties, can be seen [56]. The analysis reveals the ductile behaviour of the basalt-recycling system, which facilitates alternative failure progression in contrast with the basalt–epoxy samples, which exhibited a relatively brittle failure behaviour.

The microscopic investigation revealed certain indications of relationships between the failure mechanism and the matrix type when subjected to tensile or compressive loading states. A progressive failure mechanism, a prevalent characteristic observed in basalt–Recyclamine composite, possessed marginally reduced tensile properties and facilitated its enhanced ductile response under compression testing.

## 4. Summary and Conclusions

The mechanical performance and viability of a fully recyclable and sustainable fiber-reinforced composite material, basalt–Recyclamine (BR), were compared with a conventional sustainable but non-recyclable composite, basalt–epoxy (BE). The performances of the two composites were assessed under tensile and compressive loading states. The predictive accuracy of several theoretical models was examined. Two microscopy techniques were used to explore the composites’ microstructural characterization and failure modes. The objective was to determine the viability of basalt Recyclamine composite as a viable, effective, and sustainable option for structural applications. The study focused on evaluating the performance of each composite under tensile and compressive loading conditions.

- The tensile tests indicated BE had 18.6% greater tensile strength than BR. The difference in tensile modulus values was only 9%, reflecting the comparable stiffness of BR.
- Interestingly, BR performed impressively under compression, demonstrating slightly higher strength compared to BE (by 1.6%), but with much greater stiffness, 14.6% higher than BE. More impressively, the BR system exhibited impressive ductility with 3.9% strain capacity compared to 1.1% for basalt–epoxy, indicating higher damage tolerance and energy absorption potential of this composite system.
- SEM and digital microscopy observations at the microscopic level revealed different failure modes for the two systems. Most BE specimens demonstrated more progressive failure accompanied by a dominant fiber pull-out mechanism, whereas BE showed a more brittle fracture response. These observations corroborate well with the different mechanical properties reflective of the fiber–matrix interface mechanism.
- Theoretical prediction based on micromechanical models had reasonably good predictive ability in the case of elastic properties. The Back-out Factor method and Military Handbook technique predicted closer results to the experimental values. However, the results showed that the ideal assumptions made in their development stages would render them better suited to aerospace-grade composites (i.e., fabricated with prepreg in an autoclave), rather than composites fabricated by vacuum-assisted hand layup technique.
- Comparison with the results obtained for similarly processed composites published in the literature agreed well with the results reported in the present study. The comparison also confirmed the conclusion stated in the previous paragraph.
- Statistical analysis demonstrated the test results’ reasonably good consistency when considering the coefficient of variation (CV) values, which were essentially below 10%, affirming the reliability of the measured properties for both composite systems.
- The basalt–Recyclamine composite system has shown promising potential for industrial applications by offering satisfactory mechanical performance and clear environmental benefits compared to its traditional epoxy counterpart.

In conclusion, basalt–Recyclamine composites seem to be promising, environmentally friendly, and sustainable alternatives to conventional epoxy for industrial applications. Their comparable mechanical performance to conventional systems, with their added environmental benefit, makes these composites very attractive engineering applications that adhere to ongoing stringent environmental regulations and requirements without jeopardizing safety.

## Figures and Tables

**Table 1:** Physico-mechanical properties of neat, cured Briozen YDL5557-THR9357 Epotec system.

Mixing Ratio (Resin:Hardener) (Parts by Weight)	100:27
Viscosity (mPa.s)	180–250
Pot Life (Minutes)	200–300
Curing Temperature	Room Temp + 80 °C/8 h
Tensile Strength (MPa)	75–85
Tensile Elongation (%)	4.0–6.0
Tensile Modulus (GPa)	3.0–3.5
Flexural strength (MPa)	120–140

**Table 2:** Manufacture’s material properties of cured 105 epoxy resin [38].

Materials	105 Resin and 206 Hardener
Mixing Ratio (Resin:Hardener) (Parts by Weight)	5:1
Viscosity (mPa.s)	725
Pot Life (Minutes)	20–25
Working Time	90–110
Curing Temperature	Room temp/10–15 h, full strength/1–4 days
Tensile Strength (MPa)	50
Tensile Elongation (%)	4.5
Tensile Modulus (GPa)	3.172

**Table 3:** Dimensions of the test panels.

Test Type	Panel Dimension (mm^3^)		No. of Layers of 0/90 Basalt Fabric
	Basalt–Epoxy	Basalt–Recyclamine^®^	
Tensile	280 × 230 × 3	280 × 230 × 2.5	8
Compression	140 × 150 × 3.7	140 × 150 × 3.7	10

**Table 4:** Average tensile strength and modulus of elasticity of the two composite specimens.

Material	Tensile Properties	Avg. Value	Standard Deviation	Coeff. Var.
Basalt–epoxy	Strength (MPa)	464	25.9	5.6
	Strain (mm/mm)	0.024	0.0038	15.7
	Elastic Modulus (GPa)	22.0	2.0	8.7
Basalt–Recyclamine	Strength (MPa)	390.9	30.7	7.9
	Strain (mm/mm)	0.019	0.0037	19.9
	Elastic Modulus (GPa)	20.2	0.8	4.1

**Table 5:** Summary of compressive strength and modulus values for both composite systems.

Material	Compressive Property	Avg. Value	Standard Deviation	Coeff. Var.
Basalt–epoxy	Strength (MPa)	233.9	16	6.9
	Strain (mm/mm)	0.011	0.001	8.6
	Modulus (GPa)	24.0	0.6	2.5
Basalt–Recyclamine	Strength (MPa)	237.7	15.1	6.3
	Strain (mm/mm)	0.039	0.011	29.2
	Modulus (GPa)	27.5	1.5	5.6

**Table 6:** Comparison of the experimental and theoretical results for the tensile and compressive responses of the basalt–epoxy composite.

Back-Out Factor (BoF) = 1.5						
Stress State	Elastic Moduli			Strength (σ)		
Tensile	E_11_, RoM (GPa) 43.8		E_22_, H-Tsai (GPa) 11.9	σ_11_, BoF (MPa) 703.13	σ_11_, Simplified RoM and CLPT (MPa) 1002.2	σ_11_, MIL Alter. (MPa) 923.9
Compressive	E_11_, Exp. (GPa) 30.42	E_11_, BoF (GPa) 43.2	σ_11_, Exp. (MPa) 254.9	σ_11_, BoF (MPa) 354.5	σ_11_, Simplified RoM and CLPT (MPa) 504.9	σ_11_, Calc.—MIL Alter. (MPa) 427.1

**Table 7:** Comparison of the experimental and theoretical results for the tensile and compressive responses of the basalt–Recyclamine composite.

Back-Out Factor (BoF) = 1.5						
Stress State	Elastic Moduli			Strength (σ)		
Tensile	E_11_, RoM (GPa) 41.4		E_22_, H-Tsai (GPa) 12.2	σ_11_, BoF (MPa) 575.3	σ_11_, Simplified RoM and CLPT (MPa) 813.1	σ_11_, MIL Alter. (MPa) 798.7
Compressive	E_11_, Exp. (GPa) 29.23	E_11_, BoF (GPa) 48.3	σ_11_, Exp. (MPa) 274.9	σ_11_, BoF (MPa) 349.8	σ_11_, Simplified RoM and CLPT (MPa) 494.5	σ_11_, Calc.—MIL Alter. (MPa) 357.4

## Data Availability

The original contributions presented in this study are included in the article. Further inquiries can be directed to the corresponding author.
